# Specific Anti-SARS-CoV-2 Humoral and Cellular Immune Responses After Booster Dose of BNT162b2 Pfizer-BioNTech mRNA-Based Vaccine: Integrated Study of Adaptive Immune System Components

**DOI:** 10.3389/fimmu.2022.856657

**Published:** 2022-03-24

**Authors:** Rosalia Busà, Maria Concetta Sorrentino, Giovanna Russelli, Giandomenico Amico, Vitale Miceli, Monica Miele, Mariangela Di Bella, Francesca Timoneri, Alessia Gallo, Giovanni Zito, Daniele Di Carlo, Pier Giulio Conaldi, Matteo Bulati

**Affiliations:** ^1^ Research Department, Mediterranean Institute for Transplantation and Advanced Specialized Therapies (IRCCS ISMETT), Palermo, Italy; ^2^ Department of Laboratory Medicine and Advanced Biotechnologies, Mediterranean Institute for Transplantation and Advanced Specialized Therapies (IRCCS ISMETT), Palermo, Italy; ^3^ Department of Regenerative Medicine, Ri.MED Foundation, Palermo, Italy

**Keywords:** SARS-CoV-2, mRNA vaccine, BNT162b2, booster dose, B cells, T cells

## Abstract

Severe acute respiratory syndrome coronavirus 2 (SARS-CoV-2), which causes coronavirus disease 2019 (COVID-19), is modifying human activity all over the world with significant health and economic burden. The advent of the SARS-CoV-2 pandemic prompted the scientific community to learn the virus dynamics concerning transmissibility, epidemiology, and usefulness of vaccines in fighting emerging health hazards. Pieces of evidence suggest that the first and second doses of mRNA vaccines induce a significant antibody response in vaccinated subjects or patients who recovered from SARS-CoV-2 infection, demonstrating the importance of the previously formed memory. The aim of this work has been to investigate the effects of BNT162b2 Pfizer-BioNTech mRNA-based vaccine booster dose in a cohort of 11 uninfected immunocompetent (ICs), evaluating the humoral and cellular responses, with more carefulness on memory B and T cells. Our findings underscore the potential benefit of the third dose of mRNA vaccine on the lifespan of memory B and T cells, suggesting that booster doses could increase protection against SARS-CoV-2 infection.

## Introduction

Severe acute respiratory syndrome coronavirus 2 (SARS-CoV-2), which causes coronavirus disease 2019 (COVID-19), is modifying human activity all over the world with significant health and economic burden. The advent of the SARS-CoV-2 pandemic prompted the scientific community to learn the virus dynamics concerning transmissibility, epidemiology, and usefulness of vaccines in fighting emerging health hazards. In the last two years, many studies on vaccinated subjects or patients who recovered from SARS-CoV-2 infection (1–3) highlighted the formation of high amounts of specific antibodies, a sign of robust protective immune responses and memory. A conspicuous number of studies reported that humoral and cellular immunity to SARS-CoV-2 reaches the peak after one month from vaccination, and then it decreases over time (4–7). Conversely, circulating specific memory B lymphocytes reach the peak after two/three months and remain constant over 8 months (1, 2). In a recent study, it has been shown that the first dose of mRNA vaccines, either Pfizer-BioNTech BNT162b2 or Moderna mRNA-1273, induces a significant antibody response in COVID-19 convalescents compared to uninfected healthy individuals, demonstrating the importance of the previously formed memory (8). The emergence of new variants of SARS-CoV-2, such as B.1.617.2 (Delta) and B.1.1.529 (Omicron), able to improve transmissibility and/or the escape from antibody binding (9, 10), and the reduced effectiveness overtime of the Pfizer-BioNTech BNT162b2 vaccine (11–13), led to a resurgence of COVID-19 cases in individuals that had been vaccinated for more than six months. For these reasons, in Italy, from September 2021, a circular from the Ministry of Health approved the use of the third dose of the BNT162b2 vaccine as an additional dose for fragile individuals and, subsequently, for those who had been vaccinated for more than 4 months, in order to achieve an adequate level of the immune response. Incorrectly, the duration of protective immunity after vaccination is sometimes related solely to the level of specific antibodies. However, the most important protection from reinfection is due to the synergistic action of memory B cells, which produce specific antibodies in response to pathogen entry, and T cells, which play a key role by helping B cells to produce high-affinity antibodies and/or by eliminating virus-infected cells. Therefore, to reach a long-lasting immunity, a vaccine should not only induce robust antibodies production but also induce strong B- and T-cell responses (14).

In this article, we investigated, in a cohort of 11 uninfected immunocompetents (ICs) from our hospital staff, humoral and cellular responses, in terms of anti-spike-specific antibody production and specific memory B- and T-cell formation. Anti-spike IgG and IgA were detected in sera collected three weeks (T1) and nine months (T2) from the second dose, and three weeks after booster dose (T3) of the BNT162b2 mRNA vaccine. Circulating anti-spike memory B cells were analyzed by using unique sets of fluorescently labeled recombinant tetramers of the SARS-CoV-2 spike protein in combination with an extensive flow cytometry panel, at T2 and T3.

At the same time points, T-cell-mediated response was detected by using the QuantiFERON SARS-CoV-2, a whole-blood assay, which is based on the same platform as the QuantiFERON-TB Plus, currently approved for the diagnosis of tuberculosis and other several viral infections (15). Interestingly, a good correlation between cellular responses detected by QuantiFERON SARS-CoV-2 with ELISpot assays has been recently demonstrated (16, 17). We decided to perform the QuantiFERON assay because it is an easy-to-use tool and the only automatable test available to detect the T cellular responses in a microenvironment as close as possible to the physiological condition (16–19). Finally, T-cell reactivity to SARS-CoV-2 specific antigens was also evaluated by flow cytometry detection of activation-induced markers (AIMs) on both CD4+ (CD40L+CD69+ and OX-40+CD137+) and CD8+ (CD69+CD137+) T cells (20, 21) at T3. This is an alternative method that consents to detecting circulating antigen-specific T cells without using human leukocyte antigen (HLA)-multimers (22). In the cohort studied, we found a good antibody response at T1. Afterward, we observed a significant time-dependent decline of anti-SARS-CoV-2-specific IgG and IgA, which, in some cases, turned out to become a negativization, especially in the ones with the lowest response after the first vaccination cycle. Despite the decrease in specific antibodies, in all subjects studied, we showed the persistence of spike-specific memory B cells at T2. At the same time point, we did not find a significant T-cell response. As expected, at T3 the immune response is completely restored compared to that observed at T2 and even potentiates when compared to T1.

## Material and Methods

### Subjects Studied

A total of 11 uninfected immunocompetent (IC) healthy subjects (4 male and 7 female; median age 44, range 33–51), who never had positive nasopharyngeal swab (NPS) and anti-N response, were enrolled at the time of SARS-CoV-2 vaccination with the Pfizer-BioNTech BNT162b2 mRNA vaccine. Blood, PBMC, and serum samples were collected three weeks (T1) and nine months (T2) after the second dose and three weeks after the booster dose (T3) for the analysis of humoral and cellular immune response. The study was approved by the IRCCS-ISMETT Institutional Research Review Board (IRRB 00/21) and by the Ethic Committee of ISMETT, and all enrolled individuals signed the written informed consent form.

### Detection of SARS-CoV-2 Antibodies

Sera samples from subjects enrolled were used to detect anti-spike immunoglobulin. The chemiluminescent immunoassay (CLIA) LIAISON^®^ SARS-CoV-2 S1/S2 IgG (DiaSorin S.p.A.) was used for the quantitative detection of IgG antibodies to S1 and S2 fragments of the viral surface spike protein in the human serum. The test was used on the fully automated LIAISON^®^ XL Analyser (DiaSorin S.p.A.). The SARS-CoV-2 S1/S2 IgG antibody concentrations were expressed as arbitrary units (AU/ml) and the results were graded as follows: Negative (< 12.0 AU/ml), Equivocal (12.0–15.0 AU/ml), and Positive (> 15.0 AU/ml). The analytical performance of the assay has a good correlation to the detection of neutralizing antibodies [94.4% positive agreement to Plaque Reduction Neutralization Test (PRNT90)] and high sensitivity (95.4%) and specificity (98.6%) to ensure accurate results.

The anti-SARS-CoV-2 IgA (EUROIMMUN, Perkin Elmer Company) is an enzyme-linked immunoassay (ELISA) intended for the semiquantitative detection of IgA antibodies to S1 fragments of the viral surface spike protein, in the human serum and plasma. The test was used on the fully automated EUROIMMUN Analyzer I (EUROIMMUN, Perkin Elmer Company). The anti-SARS-CoV-2 ELISA IgA antibody concentrations were expressed as the ratio from the extinction of the sample and that of the calibrator and the results were graded as follows: Negative (< 0.8 Ratio), Equivocal (≥ 0.8 to 1.1 Ratio) and Positive (> 1.1 Ratio) With regard to performance analytical of the assay, good sensitivity (between 88.3% and 96.9%, depending on the time the sample was taken) together with high specificity (98.3%) ensures accurate results. 

In order to exclude asymptomatic infection during the overall period of follow-up, anti-N response was determined on the ARCHITECT Quant test (Abbott) using the chemiluminescent assay anti-SARS-CoV-2-N-domain CMIA (IgG and IgM) (Abbott) and SARS-CoV-2 anti-N ELISpot (see ELISpot paragraph) at T3 (data not shown).

### Isolation and Quantification of SARS-CoV-2-Specific B Cells

The venous blood of ICs was collected in K3EDTA tubes (Greiner Bio-One GmbH, Kremsmünster, Austria). Peripheral blood mononuclear cells (PBMCs) were isolated by density gradient centrifugation on Lympholyte Cell Separation Media (Cedarlane Laboratories Limited, Burlington, ON, Canada). Afterward, CD19+ B cells were separated from PBMCs by immune-magnetic sorting using anti-CD19 magnetic microbeads (REAlease CD19 MicroBeads Kit, Miltenyi Biotec, Auburn, CA, USA). The CD19+ B cells obtained from immune-magnetic sorting displayed a purity yield higher than 98%, which was determined by flow cytometry analysis. The isolated fraction was stained with SARS-CoV-2 spike B Cell Analysis Kit (ref. 130-128-022, Miltenyi Biotec, Auburn, CA, USA) to quantify the SARS-CoV-2-specific B cells at T2 and T3 following the manufacturer’s instructions. Samples were acquired by a MACSQuant Cytometer (Miltenyi Biotec, Auburn, CA, USA) and analyzed with the Kaluza Version 2.1.1 software (Beckman Coulter, CA, USA).

### QuantiFERON SARS-CoV-2 Assay

We evaluated the T-cell response at T2 and T3 by using the QuantiFERON SARS-CoV-2 kit (Qiagen, Hilden, Germany). This is an interferon gamma (IFN-γ) release assay, which contains heparinized antigen tubes that allow both to collect whole blood and to stimulate lymphocytes with a combination of three antigen peptides specific to SARS-CoV-2 (SARS-CoV-2 Ag1, Ag2, and Ag3). The SARS CoV-2 Ag1 tube contains CD4+ epitopes derived from the S1 subunit of the spike protein; the SARS CoV-2 Ag2 tube contains CD4+ and CD8+ epitopes from the S1 and S2 subunits of the spike protein; the SARS CoV-2 Ag3 tube contains CD4+ and CD8+ epitopes from S1 and S2 plus immunodominant CD8+ epitopes derived from the whole genome. After stimulation, plasma samples were analyzed for the detection of IFN-γ (IU/ml) using an ELISA-based platform. Samples were processed following the manufacturer’s instructions (QuantiFERON SARS-CoV-2 Starter kit, ref. 626115; QuantiFERON SARS-CoV-2 Extended kit, ref. 626215; QuantiFERON ELISA, ref. 626410; Qiagen, Hilden, Germany). Elevated response was defined as a value at least 0.20 IU/ml greater than Nil (negative control used to subtract IFN-γ not derived from SARS-CoV-2-specific T-cell stimulation) (16).

### SARS-CoV-2 ELISpot Assay

T-cell responses at T3 were also evaluated by using the ELISpot assay. Briefly, PBMCs from subjects studied were isolated by density gradient centrifugation from whole blood using a cell preparation tube with sodium citrate (BD Vacutainer ^®^ CPT™), according to the manufacturer’s protocol. PBMCs were counted by using the Sysmex XN-2000™ Hematology System. IFN-γ-secreting T cells were detected by a Human IFN-γ ELISpot plus kit (MABTECH AB, Sweden), according to the manufacturer’s protocol. The assay was performed, in duplicate, stimulating 2.5x10^5^ ± 0.5x10^5^ PBMCs/ml for 20–22 h, at 37°C in a 5% CO_2_ humidified atmosphere, with 1 µg/ml overlapping peptides spanning SARS-CoV-2 Spike (Mix I and II, respectively, of 158 and 157 peptides derived from a peptide scan, 15mers with 11 aa overlap) or N protein (JPT Peptide Technologies, Germany). The PBMCs were cultured in a RPMI 1640 medium (BIOWEST), supplemented with 5% GemCell U.S. Origin Human Serum AB (BIOVIT) and 1% L-Glutamine (EUROCLONE). Negative control (RPMI + 5% Human Serum AB) and positive controls, such as anti-CD3 and CEFX pepmix (a pool of 176 known peptides from various infectious agents, JPT Peptide Technologies, Germany), were also included. The number of SARS-CoV-2-specific IFN-γ-secreting cells was measured with an ELISpot Reader (Autoimmun Diagnostika (AID) GmbH, Straßberg, Germany) by using the ELISpot Software (AID). Mean spot counts for negative control wells were subtracted from the mean of test wells to generate normalized readings; these are presented as Spot Forming Unit (SFU) per million PBMCs. To determinate the lower limit to indicate a positive response (cutoff), we considered the mean value of responses of unstimulated wells plus 2 standard deviations (SD) (cutoff=121 SFC/10^6^ PBMC).

### Activation-Induced Marker (AIM) Memory T-cell Detection

Whole-blood aliquots (60 µl), at T3, were withdrawn from Nil (negative control) and mitogen (positive control) tubes and from the three Ag tubes (20 µl each, mixed together) of the QuantiFERON SARS-CoV-2 kit (Qiagen, Hilden, Germany) before centrifugation, and stained with the following combination of anti-human fluorescent monoclonal antibodies: CD3BV786, CD4FITC, CD8PE, CD137APC, CD69BV711 (Becton Dickinson, San Jose, CA, USA), CD154(CD40L)APC-VIO770, and CD134(OX-40)PE-VIO770 (Miltenyi Biotec, Auburn, CA, USA). Pharm Lyse solution (Becton Dickinson, San Jose, CA, USA) was used to remove red blood cells. Then T cells were analyzed by using a 16-color FACS Celesta SORP flow cytometer (Becton Dickinson, San Jose, CA, USA) with the same instrument setting. At least 10^4^ cells were analyzed using the Kaluza Version 2.1.1 software (Beckman Coulter, CA, USA). Cells were gated on the forward scatter/side scatter cell gate and then on the CD3+CD4+ gate for the quantification of CD40L+CD69+ and CD137+OX-40+ SARS-CoV-2-specific CD4 T cells, and on the CD3+CD8+ gate for the quantification of CD137+CD69+ SARS-CoV-2-specific CD8 T cells.

### Statistical Analysis

Statistical analysis has been performed using GraphPad Prism 9.0 (GraphPad Software, USA). Wilcoxon matched-pairs nonparametric test, Pearson and Spearman correlation, and one-way ANOVA tests with multiple comparisons have been used according to the type of samples to compare. Statistical significance was considered at p < 0.05.

## Results

### Humoral Response Elicited by Booster Dose of Pfizer-BioNTech BNT162b2 mRNA Vaccine

**Figure 1:**
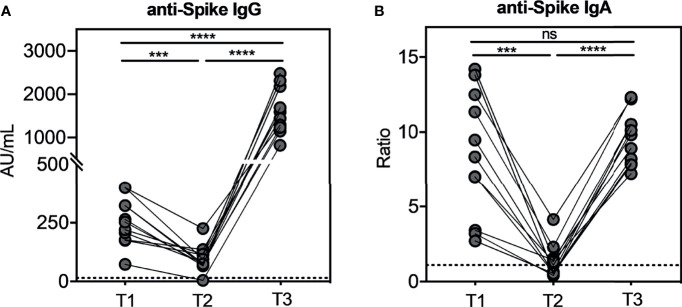
Kinetic of total anti-SARS-CoV-2 IgG and IgA serum antibodies levels in seronegative recipients (ICs n=11) of Pfizer-BioNTech BNT162b2 mRNA-based vaccination. The evaluation of both serum antibodies was conducted at three weeks (T1) and nine months (T2) after the second dose, and three weeks after the booster dose (T3). In Figure (A) anti-SARS-CoV-2 S1/S2 IgG levels and in (B) anti-SARS-CoV-2 S1 IgA levels. The dotted lines correspond to IgG (> 15.0 AU/mL) and IgA (>1.1 Ratio) cut-off, respectively. The significance was determined using Tukey’s multiple comparisons test. One-way ANOVA, ***p=0.0002; ****p<0.0001, ns, not significant.

We measured anti-SARS-CoV-2-specific antibodies, in particular IgG and IgA, by using LIAISON^®^ SARS-CoV-2 S1/S2 CLIA (DiaSorin S.p.A.) for the former and ELISA (EUROIMMUN) for the latter. At T1, the median value of specific IgG was 253 AU/ml (SEM=30.23), and it significantly decreased (p=0.0003) to 80.40 AU/ml (SEM=16.48) at T2, while at T3, we observed that the specific IgG significantly increased reaching the median value of 1,590 AU/ml (SEM=156.5) in 100% of the subjects (**Figure 1A**). Interestingly, we recorded a 20-fold increase between T3 and T2 (p<0.0001) and an 8-fold increase between T3 and T1 (p<0.0001). These results demonstrate the efficacy of the third dose to boost antibody response against SARS-CoV-2. As depicted in **Figure 1B**, we observed the same trend concerning specific IgA. Indeed, the median ratio was 8.35 (SEM=1.27) at T1, which significantly decreased to 1.10 (SEM=0.33) at T2 (p<0.0001). At T3, specific IgA had a significant upsurge (p<0.0001) compared to T2 reaching a median value of the ratio of 9.8 (SEM=0.52) compared to that observed at T1. These results suggest that the third dose restored the IgA response against SARS-CoV-2.

### SARS-CoV-2-Specific Memory B Cells Persist After Nine Months From the 2nd Dose and Increased After the Booster Dose

**Figure 2:**
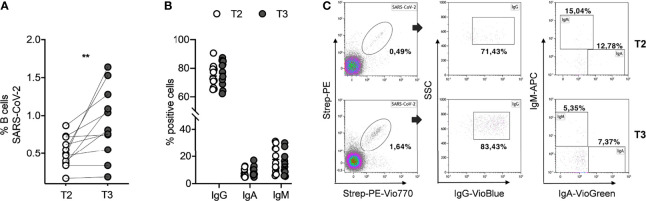
SARS-CoV-2-specific memory B cells in seronegative recipients (ICs n=11) of Pfizer-BioNTech BNT162b2 mRNA-based vaccination before (T2) and after (T3) the booster dose. **(A)** Percentage (%) of SARS-CoV-2 specific B cells before and after the booster dose. **(B)** Comparison amid T2 and T3 of percentage (%) positive cell to surface immunoglobulin isotypes, IgG, IgA, and IgM. **(C)** Representative flow cytometry plots (one subject) showing memory B-cell subpopulations. The significance was determined using Wilcoxon matched-pairs test **(A)** and Sidak’s multiple comparisons test **(B)**, one-way ANOVA, **p=0.0021.

To evaluate the long-term persistency of SARS-CoV-2-specific memory B cells after the second dose, and their kinetics after the booster dose, we assessed the identification and the characterization of these cells in all subjects studied. The SARS-CoV-2-specific B cells were characterized using a SARS-CoV-2-biotinylated-recombinant protein and two distinct fluorescently labeled streptavidin conjugates to make a spike tetramer solution. SARS-CoV-2-specific B cells were evaluated by flow cytometry in all 11 samples for the expression of markers for memory B cells (CD27) and cell surface immunoglobulin isotypes, such as IgG, IgA, and IgM, at T2 and T3. Despite a very significant reduction of circulating anti-spike antibodies (**Figures 1A, B**), we found that the percentage of SARS-CoV-2-specific memory B cells detected at T2 in our vaccinated subjects is comparable to current data in literature on the non-infected vaccinated (23) and, interestingly, is also comparable to data on COVID-19 convalescent subjects (2). Indeed, as shown in **Figure 2A**, the percent of SARS-CoV-2-specific memory B cells had a median value of 0.49% (SEM=0.06%) at T2. After the booster dose (T3), this value significantly increases compared to T2 (p=0.002), reaching a median value of 0.81% (SEM=0.14%). The phenotype analysis of spike-specific memory (CD27+) B cells reveals that most of these cells express IgG (median=72.73%, SEM=2.38% at T2; median=77.46%, SEM=2.66% at T3), and the remnants expresses IgA (median=6.29%, SEM=0.91% at T2; median=7.37%, SEM=1.10% at T3) or IgM (median=15.04%, SEM=2.84% at T2; median=12.30%, SEM=2.59% at T3) at both time points, without any significant differences (**Figure 2B**). **Figure 2C** depicts a representative flow cytometry analysis used to analyze the memory B-cell subpopulations, and **Supplementary Figure 1** shows the complete gating strategy. Briefly, cells were gated on the forward scatter/side scatter cell gate and on 7AAD-negative live cells. Subsequently, CD19+ total B cells were gated on CD27+ memory subsets and then on double-positive streptavidin conjugates for the quantification of spike-specific B cells. On this gate, IgG, IgA, and IgM immunoglobulin surface expressions were quantified.

### Cellular Response Elicited by Booster Dose Pfizer-BioNTech BNT162b2 mRNA Vaccine

**Figure 3:**
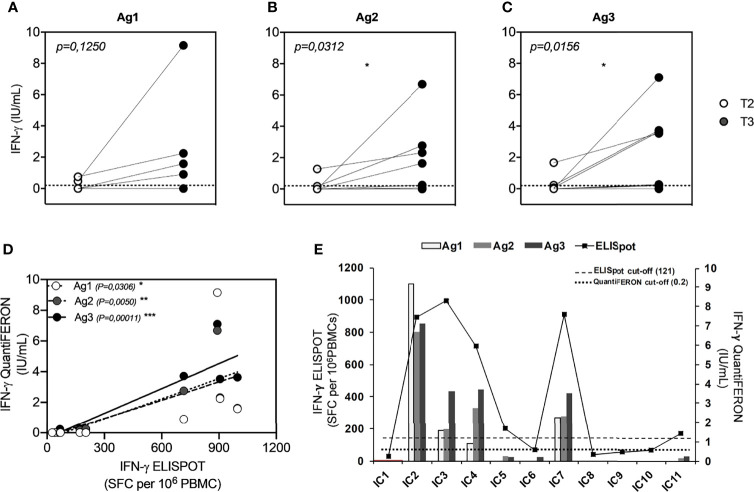
Comparison of QuantiFERON SARS-CoV-2 antigen tube (Ag minus Nil) response, express as IFN-γ (IU/ml), in seronegative subjects (ICs n=11) before (T2) and after (T3) the booster dose of the Pfizer-BioNTech BNT162b2 mRNA-based vaccine. **(A)** QFN-SARS-CoV-2 Ag1-Nil responses; **(B)** Ag2-Nil responses; **(C)** Ag3-Nil responses. The dotted lines, in each graph, correspond to IFN-γ cutoff (0.2 IU/ml). **(D)** Correlation between IFN-γ-ELISpot and IFN-γ-QFN responses of each specific antigen (Ag1 contains CD4+ epitopes derived from the S1 subunit of the spike protein; Ag2 contains CD4+ and CD8+ epitopes from the S1 and S2 subunits of the spike protein; and Ag3 contains CD4+ and CD8+ epitopes from S1 and S2, plus immunodominant CD8+ epitopes derived from the whole genome). **(E)** Graphic comparison of IFN-γ-ELISpot and IFN-γ-QFN responses of all subjects studied. The significance was determined using Wilcoxon matched-pairs test, *p = 0.0332 **(A–C)** and Pearson correlation, *p=0.0332; **p=0.0021; ***p=0.0002.

**Figure 4:**
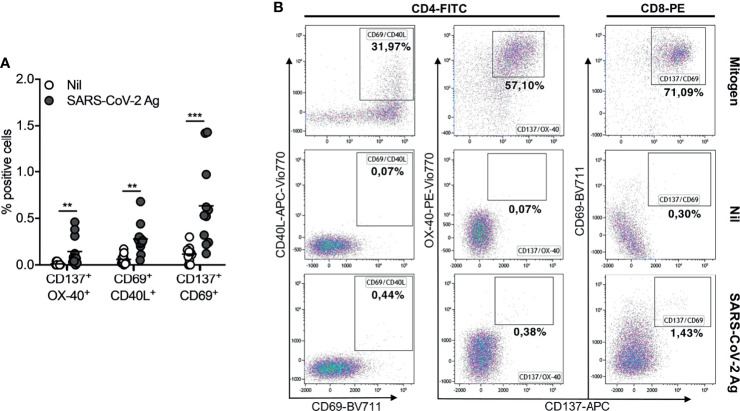
Activation-induced memory (AIM)-T cell detection in seronegative recipients (ICs n=11) of Pfizer-BioNTech BNT162b2 mRNA-based vaccination at T3. **(A)** Comparing of percentage (%) of AIM-T cells in Nil tubes to SARS-CoV-2 antigen using specific marker CD137/OX-40, CD69/CD40L, CD137/CD69 (CD40L is uniquely expressed on activated CD4 T cells; CD69 is an activation marker of both CD4 and CD8 T cells; CD137 and OX-40, both belonging to the TNF receptor superfamily, are also markers of Ag-specific CD8 and CD4 T cells, respectively. In combinations, CD137/OX-40 and CD69/CD40L identify Ag-specific CD4 T cells, while CD137/CD69 identifies Ag-specific CD8 T cells). **(B)** Representative flow cytometry plots (one subject) showing AIM-T cell subpopulations stimulated or not (Nil) with SARS-CoV-2 antigen and the positive control (Mitogen). The significance was determined using Wilcoxon matched-pairs test, **p = 0.0021; ***p = 0.0002.

To investigate whether the booster dose of the BNT162b2 Pfizer-BioNTech mRNA vaccine elicits a T-cell response to SARS-CoV-2, we performed a new IFN-γ release assay (QuantiFERON SARS-CoV-2 kit) on the whole blood of all subjects studied at T2 and T3. At T2, we showed a T-cell response, in terms of IFN-γ production, in 2/11 (18%) for Ag1 (**Figure 3A**), 2/11 (18%) for Ag2 (**Figure 3B**), and 2/11 (18%) for Ag3 (**Figure 3C**). A significant IFN-γ response in Ag2 (p=0.031) and Ag3 (p=0.016) tubes was observed at T3, compared to T2, whereas no significant responses were observed in the Ag1 tube. In particular, at T3, 4/11 (36%), 5/11 (45%), and 7/11 (64%) subjects overcame the IFN-γ cutoff (0.2 IU/ml) for Ag1, Ag2, and Ag3, respectively. Moreover, we compared IFN-γ production by both QuantiFERON and ELISpot assays. Spearman’s statistical correlation analysis revealed a positive correlation between the SARS-CoV-2 ELISPOT and SARS-CoV-2 QuantiFERON assay results. As shown in **Figure 3D**, we found significant correlations between ELISpot and Ag1 (r=0.65, p=0.03), Ag2 (r=0.78, p=0.005), and Ag3 (r=0.91, p=0.0001). These results suggest that about half of the subjects studied did not develop a good T-cell response after the booster dose (**Figure 3E**). Lastly, to define SARS-CoV-2 memory T cells, we detected on the blood coming from Ag1, Ag2, and Ag3 Quantiferon tubes the frequency of AIM-T cells. Surprisingly, we found that, at T3, all subjects showed the presence of AIM-T cells (**Figure 4A**), namely, CD40L+CD69+ (median=0.26%, SEM=0.05%; p=0.002) and CD137+OX-40+ (median=0.07%, SEM=0.05%; p=0.002) for CD4+ T cells, and CD137+CD69+ (median=0.53%, SEM=0.14%; p=0.001) for CD8+ T cells, indicative of the presence of SARS-CoV-2-specific memory T-cell compartment (22). **Figure 4B** depicts a representative flow cytometry analysis used to analyze the memory T-cell subpopulations, including the positive control (Mitogen), and **Supplementary Figure 2** shows the complete gating strategy.

## Discussion

In this study, we provided an analysis of the adaptive immune response elicited by Pfizer-BioNTech mRNA BNT162b2 vaccination in a cohort of 11 uninfected ICs. The participants have completed the anti-SARS-CoV-2 vaccination cycle, with the two recommended doses, in January 2021 and received the booster dose between October and November 2021. Three weeks after the first vaccination cycle, all subjects showed an optimal serological response, being positive for both SARS-CoV-2 anti-spike-specific IgG and IgA antibodies. We observed that anti-spike IgG and IgA antibodies drop over a nine-month period in all subjects studied. Nevertheless, about 90% (10/11) and 63% (7/11) of the participants maintained, respectively, a positive IgG and IgA value. Although serum antibodies are the most used biomarker for the evaluation of vaccine efficacy, it emerged that they are not reliable markers able to predict the immune response outcome in response to SARS-CoV2 vaccination. It is well known that serum antibody decline normally occurs in every vaccination due to the decay of short-lived plasma cells (24). The effectiveness of a vaccine depends on its ability to generate a time-enduring immunological memory, which is mediated by long-lived antigen-specific memory B and T lymphocytes. Indeed, the quantitative and qualitative analysis of antigen-specific B and T cells toward a defined antigen allows to assess whether an individual has developed an adaptive immune response to a previous immunization. Essential properties for protective memory immunity are specificity and rapidity of action. On this purpose, memory B cells, differently from memory T cells, can improve their specificity due to repeated steps of selection and somatic hypermutation, which undergo in germinal centers. Moreover, different kinetics, duration, and evolution of memory B and T cells after SARS-CoV-2 infection have been recently demonstrated. Indeed, it has been shown that spike-specific memory B cells were more abundant at 6 months than at 1 month after symptom onset and persist for up to 8 months (1). Conversely, SARS-CoV-2-specific T cells declined with a half-life of 3 to 5 months (1). In our results, obtained at nine months from the second dose of BNT162b2, we report similar findings. In fact, even if anti-spike antibody response contracts, the presence of SARS-CoV-2 memory B cells in all subjects, predominantly IgG^+^, is indicative of persisting immune memory following a second dose of vaccination. A different situation arises, instead, for the T-cell effector response, as we have shown the ability to produce IFN-γ after stimulation in a small fraction of our studied population (18%) at nine months from the primary vaccine cycle. A pronounced immune response was observed following the booster dose, including a very significant increase in anti-Spike IgG and IgA, of specific memory B cells and of T-cell response. The production of serum IgA, and the presence of IgA-expressing memory B cells, after vaccination, is of great interest because this isotype is the main antibody for protection at mucosal sites, such as the upper respiratory tracts, known to be the site of SARS-CoV-2 entry. However, in order to prevent the viral invasion of the upper airways, mucosal secretory IgA is needed (25). High levels of secretory salivary IgA have been detected in COVID-19 patients (26), but very low concentration was observed in the saliva of vaccinated individuals (27), suggesting less ability of vaccine to induce mucosal immunity. Recently, Piano Mortari et al. showed the increase in salivary IgA in vaccinated healthy individuals with a positive nasopharyngeal swab (28), demonstrating the reaction to the local infection. As a matter of fact, in most cases, vaccinated subjects with a positive nasopharyngeal swab remain asymptomatic or with mild symptoms. It could be hypothesized that part of their protection may be due to the ability of specific memory B cells to migrate to inflamed mucosal tissues, in response to attracting inflammatory molecules, and locally produce IgA. Concerning the T-cell response, after the booster dose, we observed that, despite the fact that half of the subjects did not develop a T-cell effector response, all of them present SARS-CoV-2-specific AIM-T cells, demonstrating the maintenance of long-lived memory T-cell compartment. Probably, shortening time between doses could improve the effectiveness of vaccine on the T-cell response. Although promising, these results are based on a relative short follow-up period from the booster dose and in a small cohort. Further long-term studies are necessary to investigate the duration and pliability of the immune memory induced by vaccines. The integrated study of serum antibodies, memory B cells, effector T-cell response, and memory T cells should help us to understand their time-related different kinetics and duration, with the aim to improve current or future anti-SARS-CoV-2 vaccination strategies and decisions.

## Data Availability Statement

The original contributions presented in the study are included in the article/**Supplementary Material**. Further inquiries can be directed to the corresponding author.

## Ethics Statement

The studies involving human participants were reviewed and approved by the IRCCS-ISMETT Institutional Research Review Board (IRRB 00/21) and by the Ethic Committee of ISMETT. The patients/participants provided their written informed consent to participate in this study.

## Author Contributions

RB and MB: Conceptualization, methodology, resources, and writing original draft. RB, MB, and MCS: Methodology and data curation. RB, VM, MM, FT, and MDB: Formal analysis and software. RB, MB, VM, and AG: Visualization and investigation. GZ and GA: Investigation. GR: Clinical research coordinator. PGC and DDC revised the paper critically for important intellectual content. PGC: Funding acquisition. All authors have seen and approved the final draft of the manuscript.

## Funding

This research was funded by Ministero della Salute Ricerca Finalizzata Progetto COVID-2020-12371760 and Ministero della Salute a valere sui fondi Ricerca Corrente Reti 2020 (RCR-2020): Rete Tematica IRCCS—Rete Cardiologica, grant number 23670065.

## Conflict of Interest

The authors declare that the research was conducted in the absence of any commercial or financial relationships that could be construed as a potential conflict of interest.

## Publisher’s Note

All claims expressed in this article are solely those of the authors and do not necessarily represent those of their affiliated organizations, or those of the publisher, the editors and the reviewers. Any product that may be evaluated in this article, or claim that may be made by its manufacturer, is not guaranteed or endorsed by the publisher.
