# Supplementary material for: Specific Anti-SARS-CoV-2 Humoral and Cellular Immune Responses After Booster Dose of BNT162b2 Pfizer-BioNTech mRNA-Based Vaccine: Integrated Study of Adaptive Immune System Components
Source: Front Immunol. 2022 Mar 24;13:856657. doi: 10.3389/fimmu.2022.856657 (PMC8987231; doi:10.3389/fimmu.2022.856657)
Supplement: Supplementary file 1 [file DataSheet_1.pdf]

Supplementary Material

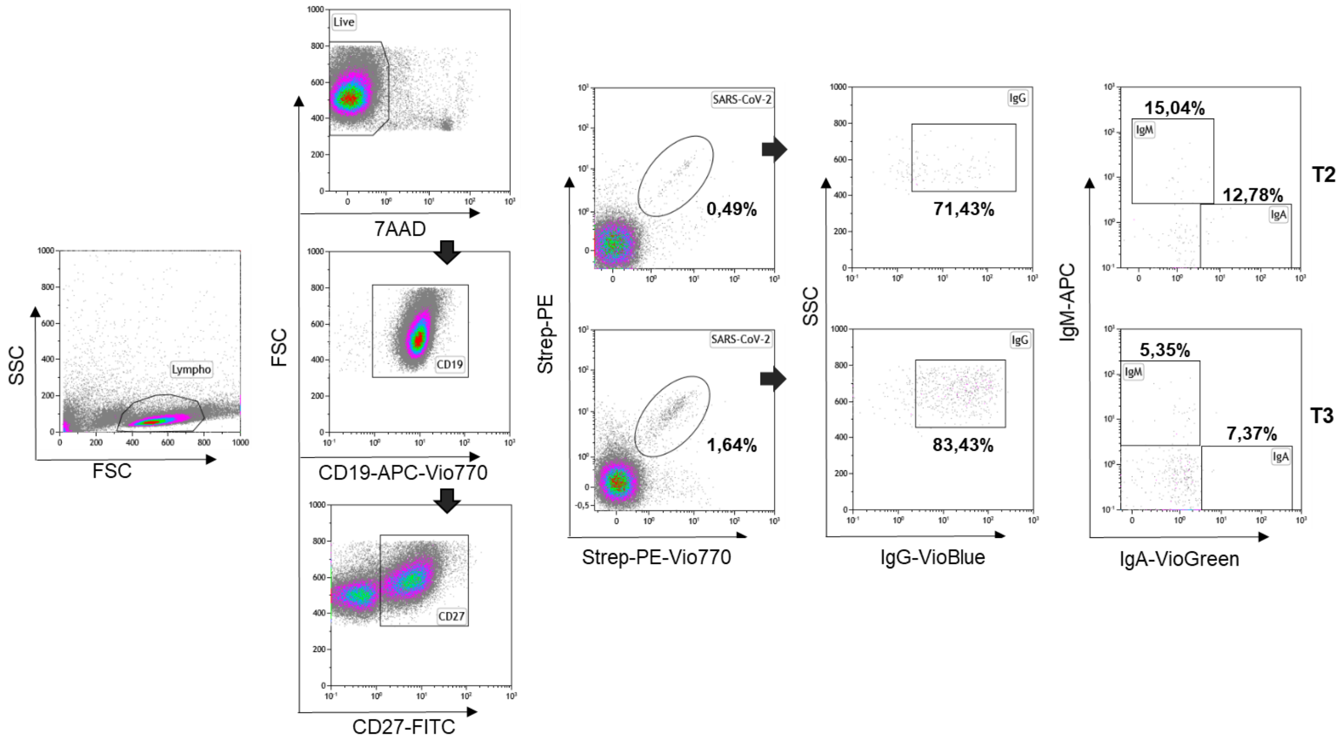

Supplementary Figure 1. Flow cytometry analysis. Representative gating strategy used to quantify memory B cells subpopulations.

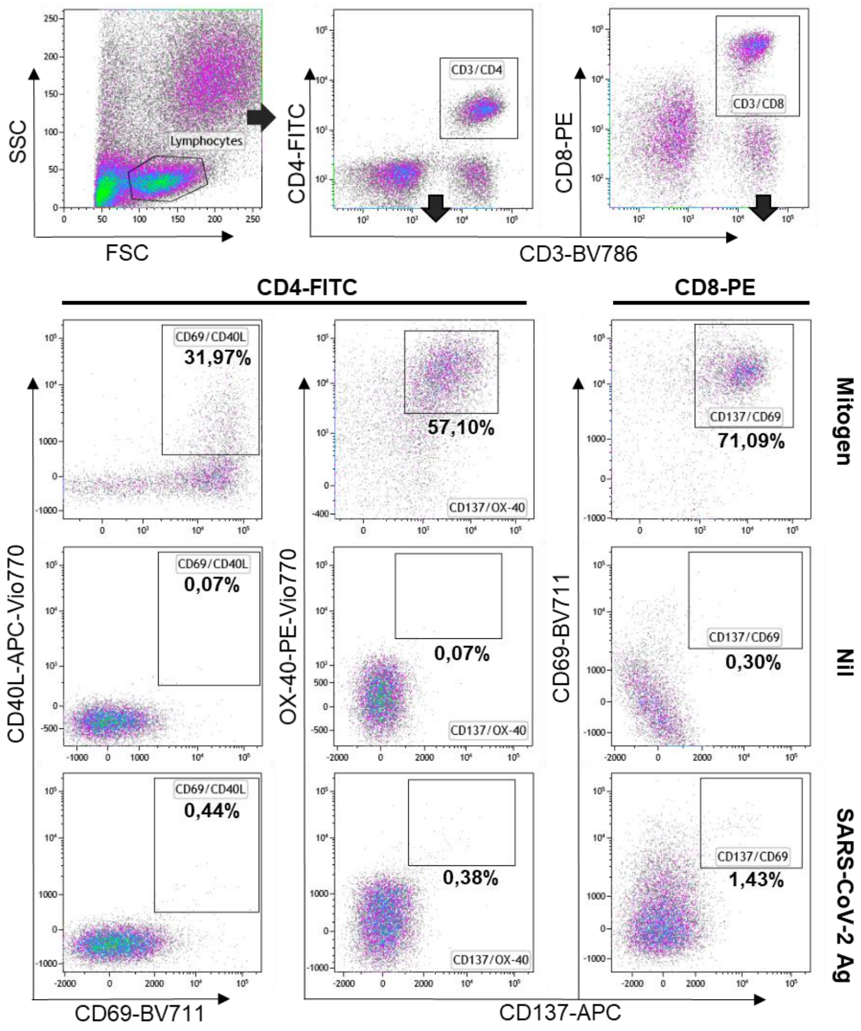

Supplementary Figure 2. Flow cytometry analysis. Representative gating strategy used to quantify memory T cells subpopulations.
